# Soil chemistry turned upside down: a meta-analysis of invasive earthworm effects on soil chemical properties

**DOI:** 10.1002/ecy.2936

**Published:** 2019-11-20

**Authors:** Olga Ferlian, Madhav P. Thakur, Alejandra Castañeda González, Layla M. San Emeterio, Susanne Marr, Barbbara da Silva Rocha, Nico Eisenhauer

**Affiliations:** 1 German Centre for Integrative Biodiversity Research (iDiv) Halle-Jena-Leipzig, Deutscher Platz 5e, 04103 Leipzig, Germany; 2 Institute of Biology, Leipzig University, Deutscher Platz 5e, 04103, Leipzig, Germany; 3 Department of Terrestrial Ecology, Netherlands Institute of Ecology (NIOO-KNAW), Droevendaalsesteeg 10, 6708 PB, Wageningen, The Netherlands; 4 National Laboratory of Genomics for Biodiversity (LANGEBIO), Center for Research and Advanced Studies of the National Polytechnic Institute (CINVESTAV), Libramiento Norte Carretera León Km 9.6, 36821, Irapuato, México; 5 Department of Biogeochemistry and Plant and Microbial Ecology, Institute of Natural Resources and Agrobiology (IRNAS), Avenida Reina Mercedes, 10, 41012, Seville, Spain; 6 Bioinformatics & Mass Spectrometry, Leibniz Institute of Plant Biochemistry, Weinberg 3, 06120, Halle (Saale), Germany; 7 Institute of Biology/Geobotany and Botanical Garden, Martin Luther University Halle Wittenberg, Am Kirchtor 1, 06108, Halle (Saale), Germany; 8 Department of Ecology, Federal University of Goiás, Esperança Avenue, Campus Samambaia, 74690-900, Goiânia, Goiás, Brazil

**Keywords:** ammonium, earthworm ecological group, element flux, exotic earthworms, nitrate, nitrification, nitrogen, nutrient cycling, pH, phosphorus, soil carbon, water content

## Abstract

Recent studies have shown that invasive earthworms can dramatically reduce native biodiversity, both above and below the ground. However, we still lack a synthetic understanding of the underlying mechanisms behind these changes, such as whether earthworm effects on soil chemical properties drive such relationships. Here, we investigated the effects of invasive earthworms on soil chemical properties (pH, water content, and the stocks and fluxes of carbon, nitrogen, and phosphorus) by conducting a meta-analysis. Invasive earthworms generally increased soil pH, indicating that the removal of organic layers and the upward transport of more base-rich mineral soil caused a shift in soil pH. Moreover, earthworms significantly decreased soil water content, suggesting that the burrowing activities of earthworms may have increased water infiltration of and/or increased evapotranspiration from soil. Notably, invasive earthworms had opposing effects on organic and mineral soil for carbon and nitrogen stocks, with decreases in organic, and increases in mineral soil. Nitrogen fluxes were higher in mineral soil, whereas fluxes in organic soil were not significantly affected by the presence of invasive earthworms, indicating that earthworms mobilize and redistribute nutrients among soil layers and increase overall nitrogen loss from the soil. Invasive earthworm effects on element stocks increased with ecological group richness only in organic soil. Earthworms further decreased ammonium stocks with negligible effects on nitrate stocks in organic soil, whereas they increased nitrate stocks but not ammonium stocks in mineral soil. Notably, all of these results were consistent across forest and grassland ecosystems underlining the generality of our findings. However, we found some significant differences between studies that were conducted in the field (observational and experimental settings) and in the lab, such as that the effects on soil pH decreased from field to lab settings, calling for a careful interpretation of lab findings. Our meta-analysis provides strong empirical evidence that earthworm invasion may lead to substantial changes in soil chemical properties and element cycling in soil. Furthermore, our results can help explain the dramatic effects of invasive earthworms on native biodiversity, for example, shifts towards the dominance of grass species over herbaceous ones, as shown by recent meta-analyses.

## Introduction

Earthworms invade terrestrial ecosystems around the globe (Hendrix and Bohlen 2002, Bohlen et al., 2004a, 2004b). Human activities have propelled the dispersal and spread of earthworms, for example, by agricultural practices, leisure (fishing), and global trade (Hendrix et al. 2008). Given their role as ecosystem engineers (Edwards 2004) and their vast potential to occupy vacant trophic niches in recipient ecosystems (Wardle et al. 2011, Eisenhauer et al. 2019), invasive earthworms have tremendous impacts on ecosystem functions, such as nutrient cycling (Lavelle et al. 2004, Bohlen et al. 2004b, Hendrix et al. 2006, Migge-Kleian et al. 2006). Recent meta-analyses further showed that the spread of invasive earthworms can dramatically alter native biodiversity, above and below the ground (Craven et al. 2017, Ferlian et al. 2018). However, studies comprehensively investigating the effects of invasive earthworms on the determinants of biodiversity (e.g., carbon and nutrient stocks) and other abiotic soil parameters are scarce. Thus, we still lack a synthetic understanding of the causes and mechanisms behind biodiversity changes with earthworm invasion.

Earthworms dominate the biomass of invertebrate fauna in the soil, and their activity can profoundly shape soil chemistry (Lavelle and Spain 2001, Edwards 2004, Eisenhauer et al. 2007, Blouin et al. 2013). They impact their environment through several actions, such as the creation of burrows, soil mixing, and removal of leaf litter (Frelich et al. 2006, Szlavecz et al. 2011). Earthworms create a dense structure of burrows that alter water infiltration rates and aeration of soil (Pérès et al. 1998, Capowiez et al. 2014). Soil aeration by invasive earthworms was found to enhance nitrification processes, which is stimulated by aerobic conditions, and fluxes of gaseous nitrogen (N) into the atmosphere (Zhu and Carreiro 1999, Araujo et al. 2004, Lubbers et al. 2013). While burrowing, earthworms secrete labile carbon (C) compounds in the form of mucus and form nutrient-rich casts (Brown 1995, Eisenhauer 2010). Burrowing activities were also found to disrupt fungal hyphae influencing the nutrient supply of plants associating with mycorrhizal fungi (Lawrence et al. 2003, Paudel et al. 2016). Moreover, soil layers are mixed and, thus, organic matter is transported to lower soil layers, resulting in a vertical redistribution of nutrients (Knollenberg et al. 1985, Eisenhauer et al. 2007). Furthermore, pH increases with earthworm invasion as earthworms transport base cations from deep mineral layers to surface layers and produce calcium carbonate granules (Hopfensperger et al. 2011).

In a recipient ecosystem, invasive earthworms are assumed to occupy vacant niches (Wardle et al. 2011), or at least have competitive predominance over native decomposers and, therefore, in the initial invasion stage, benefit from largely unlimited litter resources (Eisenhauer 2010, Eisenhauer et al. 2019). Those are removed to the extent that microhabitats and, thus, soil macro- and mesofauna in upper layers disappear (Eisenhauer et al. 2007, Eisenhauer 2010, Ferlian et al. 2018). In contrast, microfauna and microorganisms can benefit from earthworm presence, especially in earthworm burrows where earthworms secrete compounds those groups depend upon (Brown 1995, Tiunov and Scheu 1999, Tiunov et al. 2001, Savin et al. 2004). Given that soil faunal and microbial activity significantly affects the mineralization of nutrients, earthworm invasion may also impact nutrient stocks indirectly via shifts in soil faunal and microbial communities. A number of studies also found that earthworms mobilize nutrients by the enhanced comminution of organic matter in upper soil layers (Butenschön et al. 2009, Blouin et al. 2013). Interestingly, Bohlen et al. (2004b) reported both C mobilization and retention depending on the invasion stage of the ecosystem. Soil invaded by earthworms may represent a C sink in the short term driven by the mechanisms in upper soil layers mentioned above, whereas it may represent a C source in the long term because of different soil stabilization processes via casting and stable aggregate formation (Bossuyt et al. 2005, Pulleman et al. 2005, Lubbers et al. 2013). Studies on the effects of earthworm invasion on soil N stocks, however, report mixed impacts on similar time scales. Several found increased N retention and speculate that N compounds are largely locked within microbial biomass (Groffman et al. 2004, 2018). Others report an increase in N mineralization and, consequently, higher leaching and flux (Postma-Blaauw et al. 2006, Costello and Lamberti 2008, Blouin et al. 2013, Fahey et al. 2013, Lubbers et al. 2013). For phosphorus (P) cycling, the evidence is even more inconsistent, depending on the context, such as the soil type itself and the invasion stage (Suárez et al. 2004, Bohlen et al. 2004b). Studies on the effects of earthworm invasion mostly deal with total elemental concentrations or concentrations of single elemental fractions, allowing for little insight into interactions among particular soil elemental fractions. Consequently, it is difficult to make predictions on how invasive earthworms may alter whole elemental cycles. In addition, as outlined above, the effects of invasive earthworms on soil chemical properties can act in opposing directions, often dependent on the time scale, making predictions about their net effects on the ecosystem difficult.

Invasive earthworms are known to alter soil stratification and chemical gradients (Bohlen et al. 2004b, Frelich et al. 2006, Ferlian et al. 2018) by exerting different effects in different soil depths (Frelich et al. 2006, Eisenhauer 2010). Earthworm species are typically assigned to one of three ecological groups (epigeic, endogeic, and anecic; Bouché 1977). Because of their distinct life and feeding strategies and their presence in different soil layers, different earthworm invasion effects may be attributable to particular ecological groups and the soil layers they inhabit (Frelich et al. 2006, Eisenhauer 2010). For instance, anecic earthworm species build deep vertical burrows and drag high amounts of litter to lower soil layers. Thereby, they strongly contribute to the mixing of soil layers, removal of organic matter, and the redistribution of nutrients in different soil layers (Knollenberg et al. 1985). Epigeic earthworm species are found in the surface soil layers, move rather horizontally, process leaf litter at initial decomposition stages, and, along with anecic species, presumably play a major role in the flux of gaseous N from upper soil layers (Lubbers et al. 2013). Endogeic earthworms live in lower layers of the top 30 cm of the soil, ingesting large amounts of mineral soil and assimilating recalcitrant organic C resources (Ferlian et al. 2014). Through the excretion of mucus and casting, they contribute to soil aggregate stabilization processes in lower soil layers (Lavelle et al. 2004). Overall, the impact of earthworm invasion on an ecosystem thus depends on the soil layer studied, earthworm community composition, as well as on the abiotic and biotic site characteristics that determine its susceptibility to invasions. Meta-analyses have been proven to be a powerful tool to disentangle the effects earthworms exert on their abiotic and biotic environment, as was shown in Lubbers et al. (2013) and van Groenigen et al. (2014). Both of these meta-analyses confirm dramatic effects of earthworms on both soil chemistry and biology in earthworms’ native habitats. However, so far, there has not been any systematic (meta-)analysis of earthworm invasion effects on soil chemistry.

We conducted a meta-analysis on the effects of earthworm invasion on the following eight soil chemical properties: pH, water content, C, N, and P stock and C, N, and P flux. We hypothesized that (1) because of mixing and exchange of soil layer material, in organic soil, invasive earthworms deplete the stocks of C, N, and P as well as water content, whereas in mineral soil, they increase element stocks; fluxes of elements as well as pH are expected to be uniformly increased; that (2) anecic earthworms dominate the effects in both soil layers as they have the highest impact on the redistribution of organic and inorganic soil material, whereas epigeic and endogeic earthworms only have a minor impact on organic and mineral soil; that (3) different fractions of the studied elements respond differently to earthworm invasion; and that (4) the strength of the effects depends on the type of study (field observation vs. field experiment vs. lab), as study types are characterized by different exposure time of invasive earthworms and study system size.

## Methods

### Data search and selection

We compiled a data set of published data to investigate the effects of exotic earthworms on eight soil chemical properties: pH, water content, and the stocks and fluxes of C, N, and P. We conducted a search in Web of Science on September 27, 2018, using literature published between 1945 and September 2018, applying the following search string: (“lumbric*” OR “earthworm*”) AND (“invasi*” OR “exotic” OR “non-native” OR “peregrine” OR “alien” OR “introduce*”) AND (“soil NEAR/2 carbon” OR “*organic carbon” OR “soil NEAR/2 nitr*” OR “soil NEAR/2 ammoni*” OR “soil NEAR/2 phosph*” OR “soil water” OR “soil moisture” OR “soil humidity” OR “pH”). In addition, unpublished studies from doctoral theses were included in the data set. The initial search returned 109 studies. Those were screened for studies with the following inclusion criteria: (1) studies that tested the effects of exotic earthworms using an earthworm treatment/control data or regression data (earthworm biomass or abundance), if the probability was high that earthworm presence influenced the respective soil property but not vice versa; (2) studies that reported at least one of the following soil chemical properties: pH, water content, stocks or fluxes of C, N, or P; and (3) studies where control soils had been devoid of native or exotic earthworms (for studies with treatment/control data). Review, opinion, and perspectives papers were excluded from the list. The final number of studies for the meta-analysis was 40, including one doctoral thesis and two studies using regression data (Appendix S1: Table S1). We requested raw data for the two regression studies and nine further studies, as they did not report any variance or the depicted result format was not suitable for our analyses.

We collated data from the main texts, tables, and figures. We extracted means, variances, and sample sizes of treatments with (treatment) and without (control) earthworms as well as correlation coefficients of regressions between earthworm biomass/abundance and soil chemical properties and sample sizes from regression studies. Variances other than standard deviations were transformed into standard deviations. Where results were reported at several points in time, we extracted only the data corresponding to the longest experimental duration. We used the software ImageJ (Abràmoff et al. 2004) to extract data from figures. In addition, from each study, we extracted information on earthworm species studied, study type (field observation vs. field experiment vs. lab study), ecosystem/continent (continent: North America vs. Australia/Oceania; ecosystem: forest vs. grassland; note that the two covariates are entirely nested, as forest studies were only conducted in North America and grasslands studies were only conducted in Australia/Oceania), soil layer (organic vs. mineral), and the specific target response variable that was measured. These factors were used as covariates in the analyses. The final data set was comprised of four different C compounds, seven different N compounds, and 13 different P compounds (Appendix S1: Table S1).

### Data preparation

We created additional variables for each of the data sets by assigning ecological groups to the earthworm species used in the studies (after Bouché 1977), such as the presence of epigeic, endogeic, and anecic earthworm species, and ecological group richness. We further included a variable on earthworm species richness (hereafter, these five variables are called earthworm species–related covariates). We split the data into eight independent data sets according to the eight soil chemical properties, pH, water content, stock and flux of C, N, and P.

Studies that reported several soil chemical properties, used several earthworm species communities, or different soil layers contributed to the analysis with multiple observations. To account for potential dependence of observations within one study, we assigned the same study ID to those observations (see the following discussion). In total, we collected 121 observations for the analysis of soil pH, 74 observations for soil water content, 116 observations for C stock, 20 observations for C flux, 228 observations for N stock, 41 observations for N flux, 111 observations for P stock, and seven observations for the analysis of P flux (Appendix S1: Table S1).

### Data analysis

For earthworm treatment/control data, we calculated effect sizes for the effects of earthworm invasion on soil chemical properties using log-response ratio as LRR = ln(*x_i_*/*x_u_*), where *x_i_* is the mean of the invaded group, and *x_u_* is the mean of the uninvaded group. The variance of the log-response ratio was calculated using $V=S_{\text{pooled}}^{2}\left(1/(n_{i}{\left(x_{i}\right)}^{2})+1/(n_{u}{\left(x_{u}\right)}^{2})\right),$ where *S*_pooled_ is the pooled standard deviation, n_i_ is the sample size of the invaded group, and n_u_ is the sample size of the uninvaded group. For regression data, we calculated effect sizes for the effects of earthworm invasion on soil chemical properties using *z*-transformed Pearson’s correlation coefficients as *z* = 0.5 × ln((1 + *r*)/(1 − *r*)), where *z* is the *z*-transformed correlation coefficient and *r* is the correlation coefficient. The variance was calculated as *V_z_* = 1/(N − 3), where N is the sample size.

Effect sizes and variances were calculated using random-effects models (with restricted maximum-likelihood estimators) as these, in addition to sampling error, allow for across-study variability in true effect sizes (Viechtbauer 2005, Borenstein et al. 2012). The effect was significantly different from zero if 95% confidence intervals did not overlap with zero. We ran standard meta-analyses and tested for total heterogeneity of effect sizes within each model. Significant *P* values indicated heterogeneity in effects between studies when accounting for sampling error (Koricheva et al. 2013).

We explored potential publication bias in each of the eight data sets separately, using funnel plots for visual inspection (Koricheva and Gurevitch 2014), which are scatterplots of the effect sizes (*x*-axis) and standard error (*y*-axis) detecting potential publication bias based on the symmetry of the funnel shape. As a purely visual inspection is highly subjective and poorly quantitative, we, additionally, used fail-safe numbers (Rosenberg’s weighted method, Rosenberg 2005) for statistical inspection (Appendix S1: Fig. S1, Table S4) of the data, where the number of additional studies that is needed to shift the effect to a level that is not statistically significant is returned. We refrained from adjusting meta-analysis models using recent methods correcting for publication bias (Jennions et al. 2013), as this is not recommended when between-study heterogeneity is large, as in our case (Peters et al. 2007). Moreover, we investigated how much of the heterogeneity between studies is explained by the covariates (moderators) “study type,” “soil layer,” and the earthworm species–related covariates in a multilevel meta-analysis. In order to include the covariate “earthworm ecological group richness” in the model, the covariate “presence of anecic earthworm species” was removed, as these variables were collinear.

Statistical tests were only conducted on data sets that were comprised of observations from at least three studies per treatment/covariate level. Consequently, in multilevel meta-analyses for C and P flux, we had to remove covariates, such as “study type,” “soil layer,” and the presence of each of the three ecological groups, from the model. Accordingly, for water content, C stock, and N flux, the covariate study type was tested with only two levels instead of three (field observation vs. lab).

Furthermore, large sample sizes allowed us to test whether the ecosystem and continent of the study contributed to the heterogeneity between studies within the pH, N flux, and P stock data set. We used study ID as random factor in each of the models to account for the dependence of observations originating from the same study. All statistical analyses were conducted with the “metaphor” package (Viechtbauer 2010) in R (R Development Core Team 2017).

## Results

Funnel plots indicated no publication bias within each of the eight data sets, whereas fail-safe numbers pointed to potential publication bias within the data sets on C, N, and P stock (Appendix S1: Fig. S1, Table S4). The potential effects of publication bias are considered in the discussion section.

### Earthworm invasion effects on soil chemical properties

**Fig. 1:**
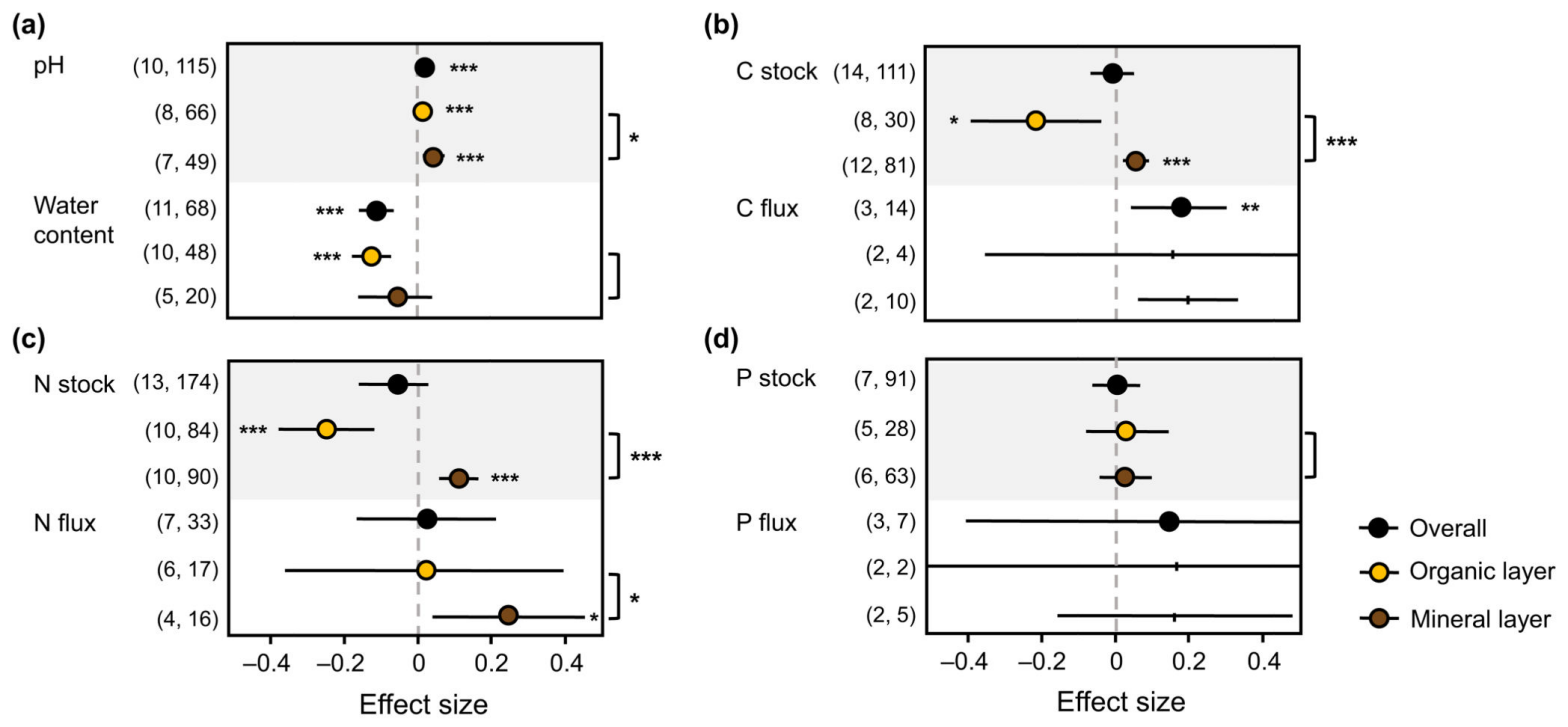
Effect sizes with 95% confidence intervals for earthworm invasion effects on (a) soil pH and water content, (b) carbon stock and flux, (c) nitrogen stock and flux, and (d) phosphorus stock and flux in total and in organic and mineral soil layers. Effects are significant when confidence intervals do not overlap with zero (indicated by asterisks, *P < 0.05, **P < 0.01, ***P < 0.001). Effect size means represented as black ticks indicate lack of studies (less than three). Values in parentheses indicate the number of studies and number of observations for the respective effect size. Asterisks outside the plot on the right indicate significant differences in effect sizes between soil layers.

Overall, water content decreased and pH and C flux increased in soils under earthworm invasion (Table 1, Fig. 1). Within each of the three properties, total heterogeneity and between-study heterogeneity was significantly low or absent (Table 1). In contrast, earthworm invasion did not significantly affect C stock, N stock and flux, and P stock and flux (Fig. 1b–d). For C, N, and P stock, between-study heterogeneity was comparably low, indicating that the variance in effect sizes between studies was low (Table 1). For N and P flux, between-study heterogeneity was high (Table 1). Multilevel meta-analysis indicated consistency of results for pH, N flux, and P stock across ecosystems/continents (note that these two covariates are not independent of each other; Appendix S1: Table S3).

Multilevel meta-analysis revealed a significant contribution of soil layer to the heterogeneity of results in most of the testable properties (Table 2). Subsequently, we ran meta-analyses for organic and mineral soil separately (Fig. 1). Effects of earthworm invasion on pH differed significantly between soil layers. Earthworm invasion increased pH in both layers, but this increase was much more pronounced in mineral than in organic soil (Fig. 1a). For C stock, N stock, and N flux, we observed opposing effects between soil layers, with negative or neutral effects in organic soil and positive effects in mineral soil (Fig. 1b, c). By contrast, effects on water content and P stock did not differ between soil layers (Table 2). Because of a lack of studies, effects on C and P flux could not be compared among soil layers (Table 2).

### Effects of earthworm ecological groups

**Fig. 2:**
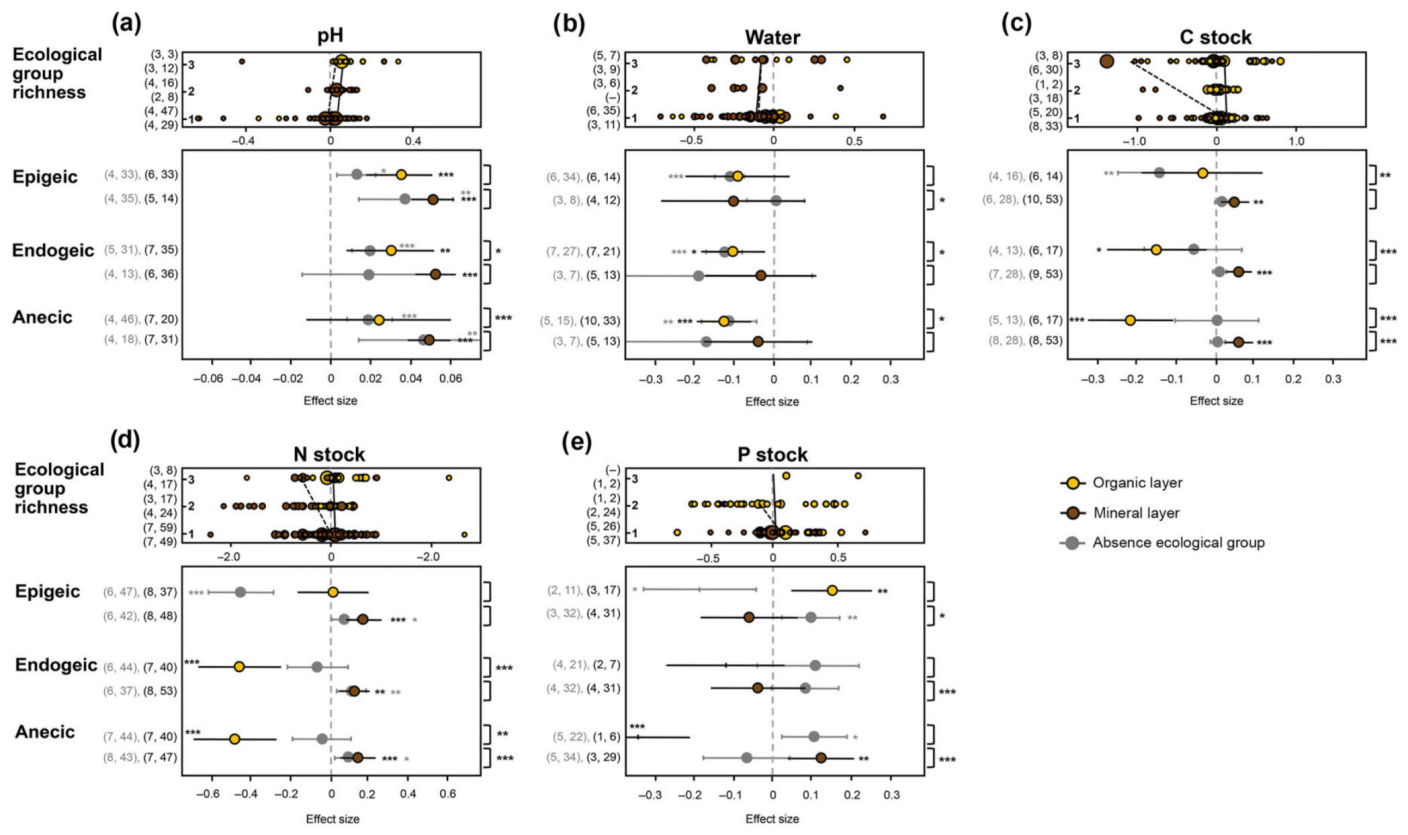
Effect sizes with 95% confidence intervals for earthworm invasion effects on soil (a) pH, (b) water content, (c) carbon stock, (d) nitrogen stock, and (e) phosphorus stock in different soil layers as affected by earthworm ecological groups richness, presence (black) and absence (gray) of epigeic, endogeic, and anecic earthworm species. Effects are significant when confidence intervals do not overlap with zero (indicated by asterisks, *P < 0.05, **P < 0.01, ***P < 0.001). Effect size means represented as ticks indicate lack of studies (less than three). Values in parentheses indicate the number of studies and number of observations for the respective effect size (presence in black, absence in gray). Asterisks outside the plot on the right indicate significant differences in effect sizes between presence and absence of the respective ecological group in the respective soil layer. The upper part of each panel is a bubble plot on earthworm ecological group richness. The size of the data points indicates the weight given to the observations. Values in parentheses next to the bubble plot indicate the number of studies and number of observations for the respective effect size (ecological group richness 1, 2, and 3) and refer to organic (top value) and mineral layer (bottom value).

Multilevel meta-analysis revealed significant contributions of earthworm species richness and ecological group richness to the heterogeneity of results for pH (Table 2). Effects of earthworm invasion significantly increased with ecological group richness in organic soil (Fig. 2a; Appendix S1: Table S2). The effects were mostly attributable to the presence of endogeic and anecic species.

Earthworm species richness significantly contributed to the effects of earthworm invasion on soil water content, but ecological group richness did not (Table 2, Fig. 2b). The presence of both endogeic and anecic species slightly contributed to the effects in organic soil, whereas the presence of epigeic species influenced the effects in mineral soil negatively.

The overall effects of earthworm invasion on C stock were mostly not mediated by earthworm species–related covariates (Table 2). However, soil layer–wise analyses revealed strong negative effects of ecological group richness in organic soil, which were mostly driven by the presence of endogeic and anecic species (Fig. 2c; Appendix S1: Table S2).

Effects of earthworm invasion on N and P stocks were significantly affected by earthworm ecological group richness, but not by species richness (Table 2). In organic soil, effects on N stock were negative and got stronger with increasing ecological group richness. On the other hand, ecological group richness only slightly influenced the effects of earthworm invasion in mineral soil (Fig. 2d; Appendix S1: Table S2). The negative effects on N stock in organic soil were mediated by the presence of endogeic and anecic species. For P stocks in organic soil, the data set was comparably small, which did not allow for tests of earthworm species–related covariates. Effects of ecological group richness on P stock in mineral soil were negative but weak (Fig. 2e; Appendix S1: Table S2). Here, epigeic and endogeic species contributed to the effect, whereas anecic species counteracted it.

### Earthworm invasion effects on soil nitrogen fractions

**Fig. 3:**
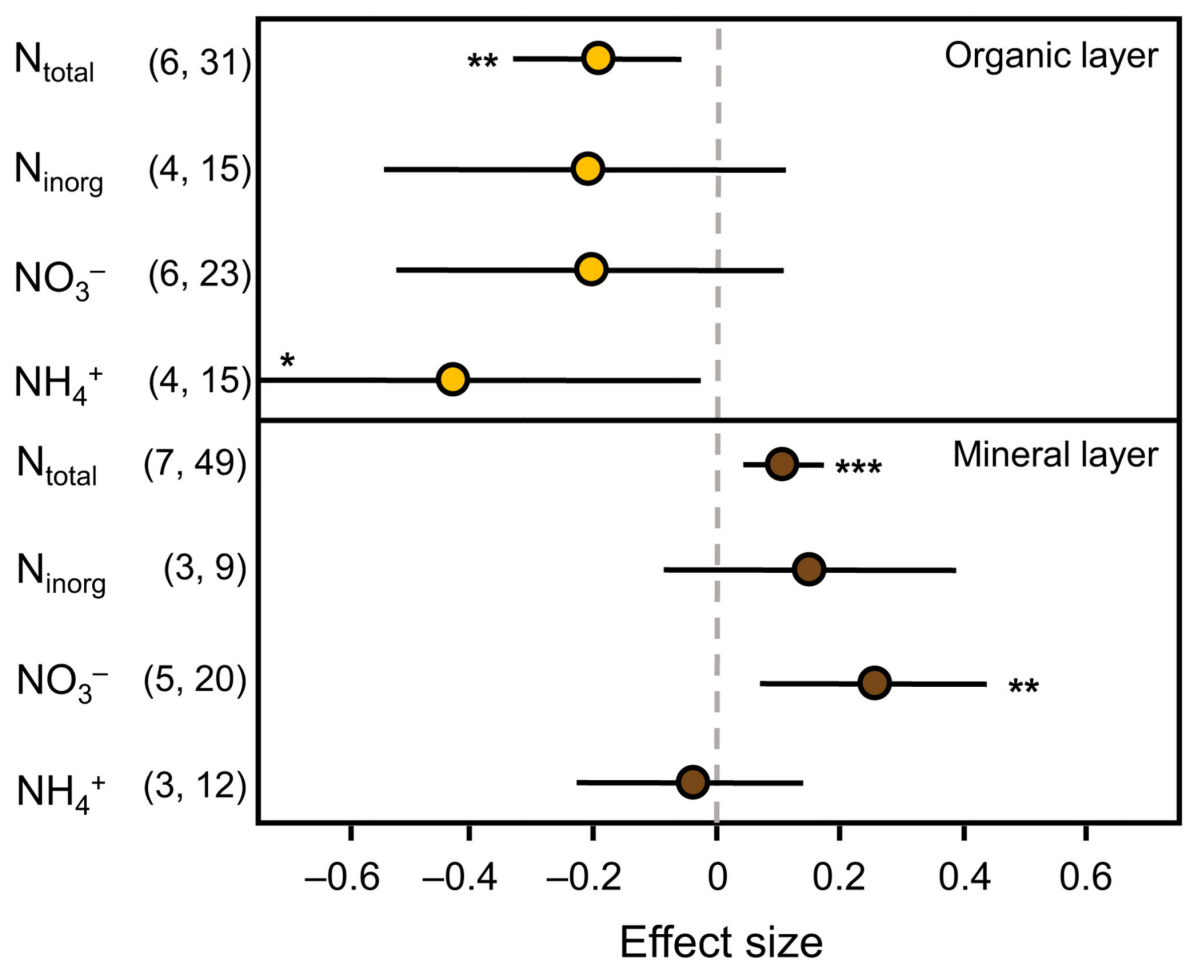
Effect sizes with 95% confidence intervals for earthworm invasion effects on soil nitrogen pools and compounds in organic and mineral soil layers. Effects are significant when confidence intervals do not overlap with zero (indicated by asterisks, *P < 0.05, **P < 0.01, ***P < 0.001). Values in parentheses indicate the number of studies and number of observations for the respective effect size. N_total_: total nitrogen, N_inorg_: inorganic nitrogen, NO_3_^−^: nitrate, NH_4_^+^: ammonium.

Earthworm invasion significantly decreased total N content in organic soil and increased it in mineral soil (Fig. 3). Inorganic N was not affected by earthworm invasion. However, in organic soil, earthworm invasion decreased ammonium and did not affect nitrate concentration, whereas, in mineral soil, ammonium was not affected but increased nitrate concentration.

### Effects of study type

**Fig. 4:**
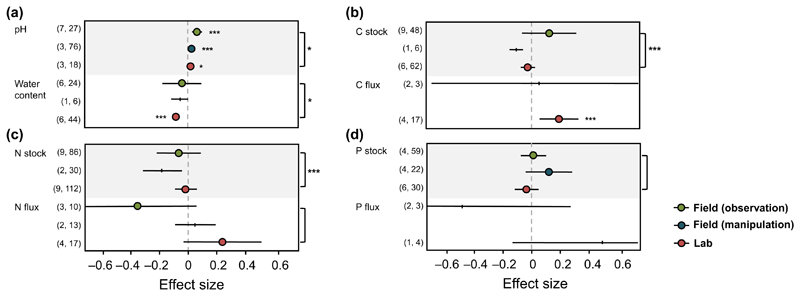
Effect sizes with 95% confidence intervals for earthworm invasion effects on (a) soil pH and water content, (b) carbon stock and flux, (c) nitrogen stock and flux, and (d) phosphorus stock and flux in different study types. Effects are significant when confidence intervals do not overlap with zero (indicated by asterisks, *P < 0.05, ***P < 0.001). Effect size means represented as black ticks indicate lack of studies (less than three). Values in parentheses indicate the number of studies and number of observations for the respective effect size. Asterisks outside the plot on the right indicate significant differences in effect sizes between study type.

Study type significantly contributed to the heterogeneity of the effects of earthworm invasion for several soil chemical properties (Table 2). Effects on pH significantly decreased from field observations, to field experiments, to lab studies (Fig. 4a). Negative effects of earthworm invasion on water content were only significant in lab studies (Fig. 4a), and the effects of earthworm invasion on C and N stocks tended to be strongest in field experiments (Fig. 4b, c). However, here, the number of studies using experimental field setups was not sufficient, which is why this finding should be treated with care. Study type did not influence the effects of earthworm invasion on P stock (Fig. 4d). The contribution of study type to the heterogeneity in the effects of earthworm invasion on C and P flux could not be tested due to a lack of data (Fig. 4b, d).

## Discussion

Our meta-analysis is the first quantitative review of earthworm invasion effects on a comprehensive set of soil chemical properties. Our key results are (1) earthworm invasion altered most of the soil chemical properties; (2) earthworm effects on soil pH and water content were consistent across soil layers, whereas the direction of effects on C, N, and P stocks, and N fractions depended on the soil layer; (3) the magnitude of earthworm invasion effects depended on the presence of endogeic and anecic species, especially in organic soil; and (4) the patterns found were consistent across ecosystems/continents, but some differed between the type of study.

### Earthworm invasion effects on soil chemistry

Overall stocks and fluxes of C and N did not respond significantly to earthworm invasion. However, separate analyses per soil layer revealed significant opposing effects of earthworm invasion, that is, negative effects in the organic and positive effects in the mineral soil. Via their burrowing activities, earthworms mix upper (nutrient-rich), with lower (nutrient-poor) soil layers with a lower proportion of organic material (Resner et al. 2011) which likely led to the detected patterns of C and N stocks. Such shifts in nutrient allocation and redistribution among soil layers may also shift soil communities in respective soil layers as shown for microbial biomass and diversity, where, accordingly, earthworm presence decreased soil microbial measures in organic soil and increased them in mineral soil (Savin et al. 2004, Ferlian et al. 2018). Shifts in microbial communities may have further implications on the distribution and availability of nutrients in soil. Moreover, it was shown previously that N content of basal soil resources is a major determinant of species richness and biomass of litter invertebrates (e.g., Jochum et al. 2017) relying on N as structural component, e.g., for the production of silk in spiders or for calcareous skeletons in arthropods (Kaspari and Yanoviak 2009). However, fail-safe numbers indicated potential publication bias in the data sets on C, N, and P stocks pointing to a careful interpretation of the findings, such as a potential lack of generality of these results.

We found lower C content in organic soil invaded by earthworms compared to uninvaded soil, but not in mineral soil. This finding is in line with previous studies, where earthworms were found to fix a considerable part of soil C in earthworm casts and stable organo-mineral complexes (Martin 1991, Scheu and Wolters 1991, Bohlen et al. 2004b, Knowles et al. 2016). Indeed, most of the studies in our meta-analysis only considered the residual (i.e., nonstable) plant available C. However, such stabilization effects may have been negligible in mineral soil as compared to mixing effects.

Our meta-analysis further revealed that soil N stock decreased in organic but increased in mineral soil. Similar as for C content, soil mixing redistributed N between organic and mineral soil. In addition, earthworms create macropores in soil that may foster gaseous losses of N into the atmosphere from upper soil layers. Enhanced soil aeration and, thus, nitrification processes, may contribute to lower N content in organic soil with earthworm invasion (Zhu and Carreiro 1999, Lubbers et al. 2013). The higher N content in mineral soil presumably led to the higher rates of N leaching found in this layer.

In contrast to C and N results, soil P stock was not significantly affected by earthworm invasion in any of the soil layers, suggesting that either invasion did not affect P cycling or that different mechanisms acted in opposing directions in the two soil layers leading to a neutral net effect. For instance, Bohlen et al. (2004b) suggested P stocks to increase in initial stages of invasion due to the increase of soil pH and in mineralization of organic matter. In later invasion stages, though, P is occluded in mineral oxides that originate from mineral soil. However, we could not disentangle such effects, as most of the studies used in our analysis did not report invasion stage. Given that effects of earthworm invasion on earthworm-free ecosystems may not be linear (Eisenhauer et al. 2019), earthworm invasion stages should be provided/estimated in future studies (see, e.g., Fisichelli et al. 2013, as an example).

For soil pH and water content, the direction of effects of earthworm invasion was consistent across soil layers, but it differed in the strength of the effect. Soil pH was higher in mineral compared to organic soil. Base cations are transported upwards from deep mineral layers by deep-burrowing anecic earthworms and may be deposited predominantly in upper mineral soil within a depth that is typically sampled (Hopfensperger et al. 2011). The stronger increase in pH in mineral soil may also be attributable to the fact that most studies using organic soil were conducted in short-term experimental field or lab settings (90–548 and 23–365 d, respectively), and studies including mineral soil were conducted in observational field settings. In the latter, the effects of earthworm invasion may be generally stronger because of their longer-term (multiyear) nature. Indeed, we found the respective pattern across study types. However, because of insufficient numbers of studies, we could not statistically test for the interaction between soil layer and study type.

Moreover, invasive earthworms decreased soil water content only in organic soil. Earthworms were shown to foster macropores in soil, which increases water infiltration rates (Pérès et al. 1998, Capowiez et al. 2014). Furthermore, soil evapotranspiration increases because of the removal of litter by incorporation into deeper soil layers and comminution by earthworms. Both effects may have led to the reduction of soil water content in organic soil.

We found consistent effects of earthworm invasion on all testable soil chemical properties (soil pH, N flux, and P stock) across ecosystems/continents (note the nestedness of the two covariates). The type of study, however, influenced the effects in most of the properties. For instance, invasive earthworms significantly decreased soil water content only in lab studies; in contrast, effects of earthworm invasion on C and N stock tended to be stronger in field observational studies compared to lab studies, presumably pointing to the importance of study duration for shifts in element distribution in soil and to the importance of soil structure affecting nutrient mobilization. To disentangle these links fully, statistical models including experimental system size and study duration will be helpful. Furthermore, it cannot be fully ruled out that a particular abiotic environment may have favored the occurrence of earthworms. Consequently, field observations may not fully separate cause and effect, and controlled field experiments are needed to infer causality (Eisenhauer et al. 2019). For instance, soil pH is determined by earthworm abundances (see above), but has also been reported to be a significant driver of earthworm abundances (Curry 1998, Fisichelli et al. 2013). However, such effects were kept at a minimum, as we only included observational field studies in the data set that investigated forests that were well known to the authors and had clear small-scale invasion fronts.

### Effects of earthworm ecological groups

Earthworm invasion effects on soil pH, water content, and element stocks depended on earthworm species richness and ecological group richness. Most of the effects on soil chemical properties strengthened with increasing ecological group richness. This potentially points to complementarity in effects because of earthworm life and feeding strategies that are different and specific for each ecological group (Bouché 1977). Such a functionally diverse earthworm community may shift the drilosphere-associated part of the soil food web, especially microbial communities which may be additional drivers of changes in soil elemental dynamics and concentrations (van der Heijden et al. 2008, Eisenhauer 2010). Moreover, the effects may be attributable to sampling effects, a common term in biodiversity–ecosystem functioning research (Tilman et al. 1997). That is, in this context, the more ecological groups are part of the earthworm community, the higher the probability is that an ecological group or species is included that has a high impact on a particular chemical property, such as *Lumbricus terrestris*, which forms deep vertical burrows, has a high burrowing activity (Edwards 2004), and represents a major part of the earthworm biomass in invaded soils (Eisenhauer et al. 2007). Indeed, our multilevel meta-analysis revealed a considerable dependence of the covariates “ecological group richness” and “presence of anecic earthworm species.” Interestingly, in organic soil, the presence of anecic and of endogeic earthworm species had significant effects on soil chemical properties. This result contradicts our hypothesis and previous assumptions that anecic species are the most crucial drivers of shifts in soil characteristics during earthworm invasion in different soil layers (Migge-Kleian et al. 2006, Groffman et al. 2015) and that the effects of endogeic species are smaller and rather restricted to mineral soil. Finally, in observational field studies, the species found in the invaded part of the site are likely a function of invasion stage with its specific soil chemical characteristics. That is, strong effects of earthworm invasion were found in studies with late invaders, that is, anecic and endogeic species, as these studies have a comparably long invasion history where impacts may have accumulated over time.

### Earthworm invasion effects on soil nitrogen cycling

Earthworm invasion decreased ammonium concentration in organic soil and increased nitrate content in mineral soil, suggesting facilitation of nitrification processes from ammonium to nitrate by earthworms. This is potentially triggered by an increase in pH and the creation of macropores, and, thus, aerobic conditions that nitrifying bacteria depend upon (Szlavecz et al. 2006, Högberg et al. 2007, Sackett et al. 2013, de Menezes et al. 2018). It is also known that ammonium and other mobile N forms increase during mineralization processes of organic matter, which is accelerated by earthworm invasion (Bohlen et al. 2004b, Hale et al. 2005, Eisenhauer et al. 2007). The shifts in ammonium and nitrate contents were related to different soil layers. This suggests that the nitrification product nitrate, which represents a more leachable form of N fraction in comparison to ammonium, may be leached into lower soil layers, where contents increased with earthworm invasion. This is in line with previous studies comparing N fractions in different soil layers (Qiu and Turner 2017).

## Conclusions

Our study provides strong evidence for significant changes in soil chemical properties and the redistribution of key elements across the soil profile promoted by earthworm invasion. Moreover, these changes depended on the earthworm community and, thus, may depend on the invasion stage of the ecosystem (Eisenhauer et al. 2019). We speculate that earthworms invading an ecosystem may have profound effects on its carbon storage potential (Groffman et al. 2004) and nutrient dynamics (Bohlen et al., 2004a, 2004b). This effect may, further, foster shifts in plant, soil microbial, and soil invertebrate communities and related ecosystem functions. Our study, therefore, complements earlier meta-analyses on the effects of invasive earthworms on plant (Craven et al. 2017), soil microbial, and invertebrate (Ferlian et al. 2018) communities that altogether corroborate the dramatic changes in ecosystem structure and function with earthworm invasion and draw a comprehensive and generalizable picture of the causes and mechanisms underlying native biodiversity change.

## Supplementary Material

Additional supporting information may be found in the online version of this article at http://onlinelibrary.wiley.com/doi/10.1002/ecy.2936/suppinfo

Appendix S1

## Figures and Tables

**Table 1:** Results of the meta-analysis for earthworm invasion effects on soil chemical properties.

	Model results				Heterogeneity			
	LRR	95% CI	SE	*P* value	τ^2^	Q	df	*P* value
pH	**0.029**	**0.021, 0.036**	**0.004**	**<0.001**	<0.001	146.219	120	0.052
Water content	−**0.100**	−**0.137**, −**0.062**	**0.019**	**<0.001**	0.011	158.303	73	<0.001
C stock	0.002	−0.057, 0.061	0.030	0.939	0.077	1056.251	114	<0.001
C flux	**0.182**	**0.051, 0.314**	**0.067**	**0.007**	0.052	48.702	19	<0.001
N stock	−0.053	−0.118, 0.012	0.033	0.111	0.184	2045.009	227	<0.001
N flux	0.038	−0.149, 0.225	0.095	0.692	0.269	497.172	40	<0.001
P stock	0.015	−0.042, 0.072	0.029	0.614	0.058	534.542	110	<0.001
P flux	0.157	−0.405, 0.720	0.287	0.583	0.494	171.574	6	<0.001

*Notes:* The section “Model results” includes effect size as log-response ratio (LLR), 95% confidence intervals (CI), standard error (SE), and *P* value. Study identity was used as random factor in the mixed-effects model. Significant effects are given in bold. The section “Heterogeneity” includes estimates of the total heterogeneity of effect size (Q), estimates of the heterogeneity between studies (τ^2^), the degrees of freedom (df), and the *P* value.

**Table 2:** Results of the meta-regression (test of moderators) for the effects of seven moderators on the magnitude of earthworm invasion effects on soil chemical properties.

		Study type (df = 2, df = 1)	Soil layer (df = 1)	EW species richness (df = 1)	EW ecological group richness (df = 1)	Presence epigeics (df = 1)	Presence endogeics (df = 1)	Presence anecics (df = 1)
pH	Q_m_	**6.943**	**4.804**	**3.893**	**14.383**	0.469	**4.752**	** 14.383**
	*P*	**0.031**	**0.028**	**0.049**	**<0.001**	0.494	**0.029**	**<0.001**
Water	Q_m_	**4.331**	2.327	**8.625**	3.030	0.165	**4.390**	3.030
content	*P*	**0.037**	0.127	**0.003**	0.082	0.684	**0.036**	0.082
C stock	Q_m_	**119.759**	**122.572**	0.003	0.010	**6.262**	3.153	1.490
	*P*	**<0.001**	**<0.001**	0.953	0.920	**0.012**	0.076	0.222
C flux	Q_m_	–	–	**9.889**	0.224	–	–	–
	*P*	–	–	**0.002**	0.636	–	–	–
N stock	Q_m_	**28.376**	**138.875**	0.052	**9.202**	0.649	**36.466**	**9.202**
	*P*	**<0.001**	**<0.001**	0.820	**0.002**	0.421	**<0.001**	**0.002**
N flux	Q_m_	**5.175**	**4.926**	2.423	0.001	**14.335**	0.029	0.001
	*P*	**0.023**	**0.027**	0.120	0.976	**<0.001**	0.865	0.976
P stock	Q_m_	2.314	1.358	1.620	**5.815**	0.033	**5.750**	**5.815**
	*P*	0.315	0.244	0.203	**0.016**	0.855	**0.017**	**0.016**
P flux	Q_m_	–	–	**4.213**	1.182	–	–	–
	*P*	–	–	**0.040**	0.277	–	–	–

*Notes:* The top value represents heterogeneity of effect sizes explained by the respective moderator (Q_m_); the bottom value represents the *P* value of the respective moderator. Study type was tested with three levels (field observation, field manipulation, and lab study) in the pH, nitrogen stock, and phosphorus stock data sets, whereas it was tested with two levels (field observation and lab study) in the water content, carbon stock, and nitrogen flux data sets due to lack of observations. Significant effects are given in bold. df: degrees of freedom, EW: earthworm.
