# Supplementary material for: Soil chemistry turned upside down: a meta-analysis of invasive earthworm effects on soil chemical properties
Source: Ecology. Author manuscript; Available in PMC 2020 Mar 3. (PMC7054046; doi:10.1002/ecy.2936)
Supplement: Appendix S1 [file EMS85394-supplement-Appendix_S1.pdf]

Supporting Information. Olga Ferlian, Madhav P. Thakur, Alejandra Castañeda González, Layla M. San Emeterio, Susanne Marr, Barbbara da Silva Rocha, and Nico Eisenhauer. 2019. Soil chemistry turned upside down: a meta-analysis of invasive earthworm effects on soil chemical properties. *Ecology.*

Appendix S1

Table S1. List of studies included in the meta-analysis and their characteristics. obs.: observation; exp.: experiment; DON: dissolved organic nitrogen; DOC: dissolved organic carbon.

Study ID	Year published	No. of observations	Earthworm species	Earthworm ecological group	Study type	Ecosystem	Continent	Biome	Chemical property	Property fraction	Data type
Burtelow et al.	1998	6	*Aporrectodea* spp.	endogeic	field (obs.)	forest	North America	temperate forest	C stock, N stock	C_{inorg}, NO_3^-, NH_4^+	Means and SD
Cameron et al.	2012	4	*Lumbricus terrestris, Dendrobaena octaedra*	anecic, epigeic	lab	forest	North America	boreal forest	N stock	total N	Means and SD
Chan et al.	2004	50	*Aporrectodea longa, Aporrectodea caliginosa, Aporrectodea trapezoides*	anecic, endogeic	field (exp.)	grassland	Australia/Oceania	temperate grassland	pH, N flux	pH, N_{inorg}	Means and SD
Chang et al.	2017	30	*Amynthas agrestis, Lumbricus rubellus, Amynthas corticis, Octolasion lacteum*	endogeic, epi-endogeic	field (exp.)	forest	North America	temperate forest	pH	pH	Means and SD
Crumsey et al.	2013	49	*Lumbricus terrestris, Aporrectodea trapezoides, Eisenia fetida*	anecic, endogeic, epigeic	lab	forest	North America	temperate forest	C stock, N stock, C flux	total C, total N, DOC	Means and SD
Crumsey et al.	2015	30	*Lumbricus terrestris, Aporrectodea trapezoides, Eisenia fetida*	anecic, endogeic, epigeic	lab	forest	North America	temperate forest	C stock, N stock, C flux	total C, total N, DOC	Means and SD
de Menezes et al.	2018	12	*Aporrectodea trapezoides*	endogeic	lab	grassland	Australia/Oceania	temperate grassland	pH, water, N stock, N flux	pH, water, NO_3^-, DON, free amino acid N, NH_4^+	Means and SD
Dempsey et al.	2013	6	*Lumbricus rubellus, Lumbricus terrestris*	anecic, epi-endogeic	field (obs.)	forest	North America	temperate forest	pH, C stock	pH, C_{org}	Means and SD
Eisenhauer et al.	2007	2	*Lumbricus terrestris, Dendrobaena octaedra, Octolasion tyrtaeum*	anecic, endogeic, epigeic	field (obs.)	forest	North America	boreal forest	pH	pH	Correlation coefficients
Eisenhauer et al.	2011	4	*Lumbricus rubellus, Aporrectodea* spp.*, Lumbricus terrestris, Octolasion tyrtaeum*	anecic, endogeic, epigeic	field (obs.)	forest	North America	temperate forest	water	water	Means and SD
Eisenhauer et al.	2012	16	*Aporrectodea caliginosa, Lumbricus rubellus, Lumbricus terrestris*	anecic, endogeic, epigeic	lab	forest	North America	temperate forest	water	water	Means and SD
Eisenhauer et al.	2012	9	*Aporrectodea caliginosa, Lumbricus terrestris*	anecic, endogeic	lab	forest	North America	temperate forest	water	water	Means and SD
Fahey et al.	2013	1	*Lumbricus terrestris, Lumbricus rubellus, Octolasion tyrtaeum, Apporectodea* spp.	anecic, endogeic, epigeic	field (obs.)	forest	North America	temperate forest	C stock	total C	Means and SD
Greiner et al.	2012	12	*Amynthas hilgendorfi, Lumbricus rubellus*	epi-endogeic, epigeic	lab, field (exp.)	forest	North America	temperate forest	N flux, P stock	N_{inorg}, exchangeable P	Means and SD
Groffman et al.	2004	20	*Lumbricus rubellus, Lumbricus terrestris, Octolasion tyrtaeum*	anecic, endogeic, epi-endogeic	field (obs.)	forest	North America	temperate forest	N stock, N flux	N_{inorg}, NO_3^-	Means and SD
Groffman et al.	2015	28	*Lumbricus terrestris, Octolasion tyrtaeum, Lumbricus rubellus*	anecic, endogeic, epi-endogeic	field (obs.)	forest	North America	temperate forest	N stock, N flux	total N, N_{inorg}, NO_3^-	Means and SD
Hale et al.	2005	18	*Lumbricus rubellus, Lumbricus* spp.*, Lumbricus terrestris, Dendrobaena* spp.*, Octolasion* spp.*, Aporrectodea* spp.	anecic, endogeic, epi-endogeic, epigeic	field (obs.)	forest	North America	temperate forest	N stock, P stock	NO_3^-, NH_4^+, PO_4^{3-}	Means and SD
Hale et al.	2008	12	*Lumbricus terrestris, Lumbricus rubellus, Dendrobaena octaedra*	anecic, epigeic	lab	forest	North America	temperate forest	N stock, P stock	NO_3^-, NH_4^+, PO_4^{3-}	Means and SD
He et al.	2018	4	*Pontoscolex corethrurus*	endogeic	lab	forest	Asia	subtropical forest	N stock	NH_4^+, NO_3^-	Means and SD
Jennings and Watmough	2016	10	*Dendrobaena octaedra, Aporrectodea turgida,*	anecic, endogeic, epigeic	field (obs.)	forest	North America	temperate forest	C stock, pH	total C, pH	Means and SD

			Aporrectodea rosea, Lumbricus terrestris								
Kim et al.	2017	32	*Octolasion cyaneum, Eisenia fetida*	endogeic, epigeic	lab	grassland	Australia/Oceania	temperate grassland	pH, water, C stock, N stock, P stock, N flux	total C, pH, water, Olsen P, total N, N_2O, NO_3^-, NH_4^+	Means and SD
Lawrence et al.	2003	2	*Lumbricus rubellus, Octolasion tyrtaeum, Lumbricus terrestris*	anecic, epi-endogeic	field (obs.)	forest	North America	temperate forest	P stock	Resin P	Means and SD
Li et al.	2002	2	*Lumbricus rubellus, Lumbricus terrestris, Amynthas hawayanus*	anecic, epi-endogeic	field (obs.)	forest	North America	temperate forest	water	water	Means and SD
Lyttle et al.	2015	12	*Octolasion* spp., *Aporrectodea spp., Lumbricus juvenile, Lumbricus rubellus, Dendrobaena octaedra, Lumbricus terrestris*	anecic, endogeic, epi-endo-anecic, epi-endogeic, epigeic	field (obs.)	forest	North America	temperate forest	C stock	total C	Means and SD
Migge (dissertation)	2001	114	*Octolasion tyrtaeum, Aporrectodea caliginosa, Lumbricus terrestris*	anecic, endogeic	field (exp.), lab	forest	North America	boreal forest	pH, water, C stock, N stock, P stock, N flux	pH, water, total C, total N, NO_3^-, NH_4^+, PO_4^{3-}	Means and SD
Muir et al.	2007	3	*Aporrectodea trapezoides*	endogeic	lab	grassland	Australia/Oceania	temperate grassland	P stock	Colwell P	Means and SD
Psarska et al.	2016	4	*Lumbricus rubellus, Dendrobaena octaedra, Aporrectodea* spp., *Lumbricus terrestris, Octolasion tyrtaeum*	anecic, endogeic, epigeic	field (obs.)	forest	North America	temperate forest	C stock	C_{org}	Means and SD
Qiu and Turner	2017	42	*Amynthas agrestis, Amynthas tokioensis*	epigeic	field (obs.), lab	forest	North America	temperate forest	C stock, N stock, P stock, C flux	total C, total N, Bray P, DOC, N_{inorg}, NH_4^+, NO_3^-	Means and SD
Roth et al.	2015	9	*Lumbricus terrestris*	anecic	lab	forest	North America	temperate forest	water	water	Means and SD
Sackett et al.	2013	6	*Dendrobaena octaedra, Dendrodrilus rubidus, Lumbricus rubellus, Aporrectodea rosea, Aporrectodea caliginosa, Lumbricus terrestris*	anecic, endogeic, epi-endogeic, epigeic	field (obs.)	forest	North America	temperate forest	pH, P flux, water, N stock, N flux, C flux	pH, DOP, NH_4^+, DON, DOC, NO_3^-	Means and SD
Saltmarsh et al.	2016	2	*Dendrobaena octaedra, Lumbricus terrestris*	anecic, epigeic	field (obs.)	forest	North America	boreal forest	pH, water	pH, water	Means and SD
Scheu and Parkinson	1994a	25	*Dendrobaena octaedra*	epigeic	lab, field (exp.)	forest	North America	boreal forest	N stock, P stock, N flux	NH_4^+, PO_4^{3-}, N_{inorg}	Means and SD
Scheu and Parkinson	1994b	11	*Dendrobaena octaedra*	epigeic	lab	forest	North America	boreal forest	N stock, P stock	NO_3^-, PO_4^{3-}, NH_4^+	Means and SD
Straube et al.	2009	36	*Lumbricus terrestris, Octolasion tyrtaeum, Dendrobaena octaedra*	anecic, endogeic, epigeic	field (obs.)	forest	North America	boreal forest	pH water, C stock, N stock	pH, water, total C, total N	Correlation coefficients
Suárez et al.	2003	52	*Lumbricus terrestris, Lumbricus rubellus, Octolasion tyrtaeum*	anecic, endogeic, epi-endogeic	field (obs.)	forest	North America	temperate forest	P stock, P flux	Occluded P, NaOH-P_o, NaOH-P_i, total P, Na-Bicarbonate-P_o, HCl-P, Resin-P, Na-Bicarbonate-P_i, DOP	Means and SD
Suárez et al.	2006	2	*Lumbricus terrestris, Lumbricus rubellus, Octolasion tyrtaeum, Aporrectodea tuberculata*	anecic, endogeic, epi-endogeic	field (obs.)	forest	North America	temperate forest	water	water	Means and SD
Whitfeld et al.	2014	4	*Lumbricus terrestris*	anecic	lab	forest	North America	temperate forest	water	water	Means and SD
Wironen and Moore	2006	14	*Aporrectodea tuberculata, Dendrobaena octaedra, Lumbricus terrestris, Lumbricus rubellus, Eisenia rosea*	anecic, endogeic, epi-endogeic, epigeic	field (obs.)	forest	North America	temperate forest	C stock, N stock	total C, total N	Means and SD
Yavitt	2015	21	*Lumbricus rubellus, Lumbricus terrestris, Octolasion tyrtaeum, Aporrectodea tuberculata*	anecic, endogeic, epi-endogeic	field (obs.)	forest	North America	temperate forest	water, pH, N stock	water, pH, NO_3^-	Means and SD
Zhang et al.	2013	2	*Lumbricus rubellus, Amynthas agrestis*	epi-endogeic, epigeic	lab	forest	North America	temperate forest	C stock	C_{org}	Means and SD

Table S2. Soil layer-wise results of the meta-regression (test of moderators) for the moderators ecological group richness, presence epigeic species, presence endogeic species, and presence anecic species on the magnitude of earthworm invasion effects on soil chemical properties (see Fig. 2, main document). The first value represents heterogeneity of effect sizes explained by the respective moderator (Q_m); the second value represents the *P*-value of the respective moderator (*df* = 1). Significant effects are given in bold. EW: earthworm.

	Moderator	Soil layer	Q_m	*P*
pH	EW ecol. group richness	organic	**13.483**	**<0.001**
		mineral	0.165	0.684
	Presence epigeics	organic	0.204	0.651
		mineral	0.131	0.718
	Presence endogeics	organic	**4.206**	**0.040**
		mineral	0.719	0.397
	Presence anecics	organic	**13.483**	**<0.001**
		mineral	0.165	0.684
Water content	EW ecol. group richness	organic	**5.756**	**0.016**
		mineral	0.216	0.642
	Presence epigeics	organic	0.406	0.524
		mineral	**3.862**	**0.049**
	Presence endogeics	organic	**4.673**	**0.031**
		mineral	2.767	0.096
	Presence anecics	organic	**5.756**	**0.016**
		mineral	0.216	0.642
C stock	Ew ecol. group richness	organic	**11.433**	**<0.001**
		mineral	**11.122**	**<0.001**
	Presence epigeics	organic	**9.372**	**0.002**
		mineral	0.049	0.825
	Presence endogeics	organic	**41.085**	**<0.001**
		mineral	3.548	0.060
	Presence anecics	organic	**11.433**	**<0.001**
		mineral	**11.114**	**<0.001**
N stock	EW ecol. group richness	organic	**9.789**	**0.002**
		mineral	**11.182**	**<0.001**
	Presence epigeics	organic	0.572	0.450
		mineral	1.361	0.243
	Presence endogeics	organic	**62.615**	**<0.001**
		mineral	0.120	0.730
	Presence anecics	organic	**9.789**	**0.002**
		mineral	**11.182**	**<0.001**
P stock	EW ecol. group richness	organic	-	-
		mineral	**22.078**	**<0.001**
	Presence epigeics	organic	-	-
		mineral	**4.351**	**0.037**
	Presence endogeics	organic	-	-
		mineral	**10.615**	**<0.001**
	Presence anecics	organic	-	-
		mineral	**22.078**	**<0.001**

Table S3. Results of the meta-regression (test of moderators) for the effects of the moderator ecosystem/continent on the magnitude of earthworm invasion effects on soil chemical properties. The first value represents heterogeneity of effect sizes explained by the moderator (Q_m); the second value represents the *P*-value of the moderator (*df* = 1).

	Q_m	*P*
pH	0.020	0.889
Water content	-	-
C stock	-	-
C flux	-	-
N stock	-	-
N flux	0.075	0.784
P stock*	0.128	0.721
P flux	-	-

* results based on two studies only.

Table S4. Fail-safe numbers (Rosenberg's weighted method; Rosenberg 2005), number of studies and comparison of fail-safe numbers with the number of studies x 5 + 10 (5*k* + 10, *k* = number of studies in the dataset) of the eight datasets on soil abiotic properties as affected by earthworm invasion. Based on the fail-safe numbers, YES indicates that no publication bias exists, NO indicates that publication bias potentially exists.

	Fail-safe-N	No. studies	Fail-safe-N > No. Studies
pH	3169	121	YES
Water content	3822	74	YES
C stock	398	116	NO
C flux	223	20	YES
N stock	518	228	NO
N flux	2209	41	YES
P stock	0	111	NO
P flux	789	7	YES

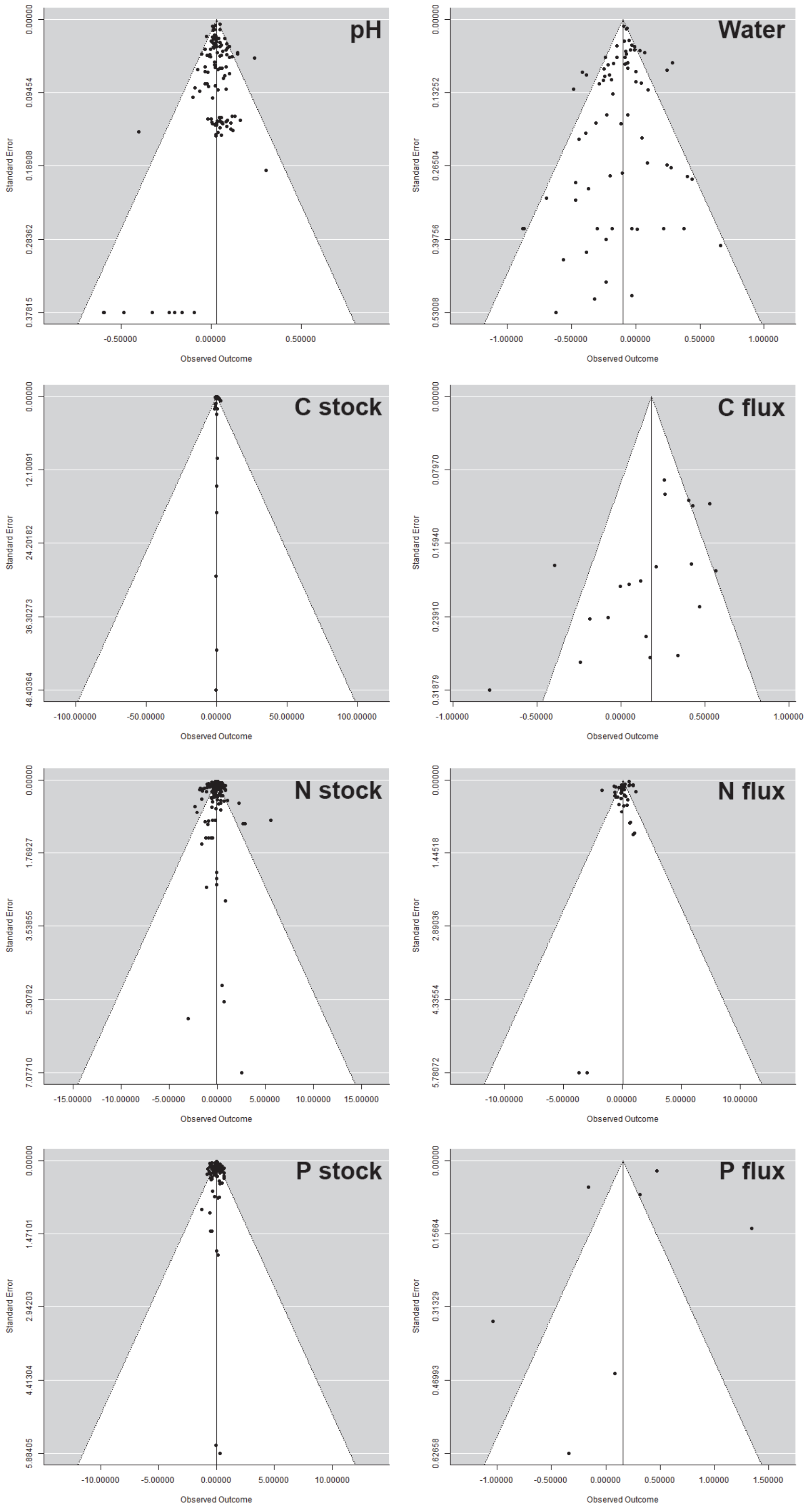

pH
Water
C stock
C flux
N stock
N flux
P stock
P flux
Standard Error
Observed Outcome

Fig. S1. Funnel plots for responses of eight soil abiotic properties to invasive earthworms. The x-axis is the effect size (log response ratio) with its standard error as the y-axis. Points in the gray area are outliers. Publication bias is present if the data distribution around the zero line is asymmetric.

References

Burtelow, A. E., P. J. Bohlen, and P. M. Groffman. 1998. Influence of exotic earthworm invasion on soil organic matter, microbial biomass and denitrification potential in forest soils of the northeastern United States. Applied Soil Ecology 9:197-202.

Cameron, E. K., M. W. Zabrodski, J. Karst, and E. M. Bayne. 2012. Non-native earthworm influences on ectomycorrhizal colonization and growth of white spruce. Ecoscience 19:29-37.

Chan, K. Y., G. H. Baker, M. K. Conyers, B. Scott, and K. Munro. 2004. Complementary ability of three European earthworms (Lumbricidae) to bury lime and increase pasture production in acidic soils of south-eastern Australia. Applied Soil Ecology 26:257-271.

Chang, C. H., K. Szlavecz, and J. S. Buyer. 2017. *Amynthas agrestis* invasion increases microbial biomass in Mid-Atlantic deciduous forests. Soil Biology and Biochemistry 114:189-199.

Crumsey, J. M., J. M. Le Moine, Y. Capowiez, M. M. Goodsitt, S. C. Larson, G. W. Kling, and K. J. Nadelhoffer. 2013. Community-specific impacts of exotic earthworm invasions on soil carbon dynamics in a sandy temperate forest. Ecology 94:2827-2837.

Crumsey, J. M., Y. Capowiez, M. M. Goodsitt, S. Larson, J. M. Le Moine, J. A. Bird, ... and K. J. Nadelhoffer. 2015. Exotic earthworm community composition interacts with soil texture to affect redistribution and retention of litter-derived C and N in northern temperate forest soils. Biogeochemistry 126:379-395.

de Menezes, A. B., M. T. Prendergast-Miller, L. M. Macdonald, P. Toscas, G. Baker, M. Farrell, T. Wark, A. E. Richardson, and P. H. Thrall. 2018. Earthworm-induced shifts in

microbial diversity in soils with rare versus established invasive earthworm populations. FEMS Microbiology Ecology 94:1-14.

Dempsey, M. A., M. C. Fisk, J. B. Yavitt, T. J. Fahey, and T. C. Balser. 2013. Exotic earthworms alter soil microbial community composition and function. Soil Biology and Biochemistry 67:263-270.

Eisenhauer, N., J. Schlaghamerský, P. B. Reich, and L. E. Frelich. 2011. The wave towards a new steady state: effects of earthworm invasion on soil microbial functions. Biological Invasions 13:2191.

Eisenhauer, N., N. A. Fisichelli, L. E. Frelich, and P. B. Reich. 2012. Interactive effects of global warming and 'global worming' on the initial establishment of native and exotic herbaceous plant species. Oikos 121:1121-1133.

Eisenhauer, N., S. Partsch, D. Parkinson, and S. Scheu. 2007. Invasion of a deciduous forest by earthworms: Changes in soil chemistry, microflora, microarthropods and vegetation. Soil Biology and Biochemistry 39:1099–1110.

Fahey, T. J., J. B. Yavitt, R. E. Sherman, J. C. Maerz, P. M. Groffman, M. C. Fisk, and P. J. Bohlen. 2013. Earthworm effects on the incorporation of litter C and N into soil organic matter in a sugar maple forest. Ecological Applications 23:1185-1201.

Greiner, H. G., D. R. Kashian, and S. D. Tiegs. 2012. Impacts of invasive Asian (*Amynthas hilgendorfi*) and European (*Lumbricus rubellus*) earthworms in a North American temperate deciduous forest. Biological Invasions 14:2017-2027.

Groffman, P. M., P. J. Bohlen, M. C. Fisk, and T. J. Fahey. 2004. Exotic earthworm invasion and microbial biomass in temperate forest soils. Ecosystems 7:45-54.

Groffman, P. M., T. J. Fahey, M. C. Fisk, J. B. Yavitt, R. E. Sherman, P. J. Bohlen, and J. C. Maerz. 2015. Earthworms increase soil microbial biomass carrying capacity and nitrogen retention in northern hardwood forests. Soil Biology and Biochemistry 87:51-58.

Hale, C. M., L. E. Frelich, P. B. Reich, and J. Pastor. 2005. Effects of European earthworm invasion on soil characteristics in northern hardwood forests of Minnesota, USA. Ecosystems 8:911–927.

Hale, C. M., L. E. Frelich, P. B. Reich, and J. Pastor. 2008. Exotic earthworm effects on hardwood forest floor, nutrient availability and native plants: a mesocosm study. Oecologia 155:509-518.

He, X., Y. Chen, S. Liu, A. Gunina, X. Wang, W. Chen, ... and X. Zou. 2018. Cooperation of earthworm and arbuscular mycorrhizae enhanced plant N uptake by balancing absorption and supply of ammonia. Soil Biology and Biochemistry 116:351-359.

Jennings, B. W., and S. A. Watmough. 2016. The impact of invasive earthworms on soil respiration and soil carbon within temperate hardwood forests. Ecosystems 19:942-954.

Kim, Y. N., B. Robinson, K. A. Lee, S. Boyer, and N. Dickinson. 2017. Interactions between earthworm burrowing, growth of a leguminous shrub and nitrogen cycling in a former agricultural soil. Applied Soil Ecology 110:79-87.

Lawrence, B., M. C. Fisk, T. J. Fahey, and E. R. Suárez. 2003. Influence of nonnative earthworms on mycorrhizal colonization of sugar maple (*Acer saccharum*). New Phytologist 157:145-153.

Li, X., M. C. Fisk, T. J. Fahey, and P. J. Bohlen. 2002. Influence of earthworm invasion on soil microbial biomass and activity in a northern hardwood forest. Soil Biology and Biochemistry 34:1929-1937.

Lyttle, A., K. Yoo, C. Hale, A. Aufdenkampe, S. D. Sebestyen, K. Resner, and A. Blum. 2015. Impact of exotic earthworms on organic carbon sorption on mineral surfaces and soil carbon inventories in a northern hardwood forest. Ecosystems 18:16-29.

Migge, S. 2001. The effect of earthworm invasion on nutrient turnover, microorganisms and microarthropods in Canadian Aspen Forest Soil. Doctoral Dissertation, TU Darmstadt, Darmstadt, Germany.

Muir, M. A., I. A. M. Yunusa, M. D. Burchett, R. Lawrie, K. Y. Chan, and V. Manoharan. 2007. Short-term responses of two contrasting species of earthworms in an agricultural soil amended with coal fly-ash. Soil Biology and Biochemistry 39:987-992.

Psarska, S., E. A. Nater, and R. K. Kolka. 2016. Impacts of invasive earthworms on soil mercury cycling: Two mass balance approaches to an earthworm invasion in a northern Minnesota forest. Water, Air, and Soil Pollution 227:205.

Qiu, J., and M. G. Turner. 2017. Effects of non-native Asian earthworm invasion on temperate forest and prairie soils in the Midwestern US. Biological Invasions 19:73-88.

Rosenberg, M. S. 2005. The file-drawer problem revisited: A general weighted method for calculating fail-safe numbers in meta- analysis. Evolution 59:464-468.

Roth, A. M., T. J. Whitfeld, A. G. Lodge, N. Eisenhauer, L. E. Frelich, and P. B. Reich. 2015. Invasive earthworms interact with abiotic conditions to influence the invasion of common buckthorn (*Rhamnus cathartica*). Oecologia 178:219-230.

Sackett, T. E., S. M. Smith, and N. Basiliko. 2013. Indirect and direct effects of exotic earthworms on soil nutrient and carbon pools in North American temperate forests. Soil Biology and Biochemistry 57:459-467.

Saltmarsh, D. M., M. L. Bowser, J. M. Morton, S. Lang, D. Shain, and R. Dial. 2016. Distribution and abundance of exotic earthworms within a boreal forest system in southcentral Alaska. NeoBiota 28:67.

Scheu, S., and D. Parkinson. 1994. Effects of earthworms on nutrient dynamics, carbon turnover and microorganisms in soils from cool temperate forests of the Canadian Rocky Mountains - laboratory studies. Applied Soil Ecology 1:113-125.

Scheu, S., and D. Parkinson. 1994. Effects of invasion of an aspen forest (Canada) by *Dendrobaena octaedra* (Lumbricidae) on plant growth. Ecology 75:2348-2361.

Straube, D., E. A. Johnson, D. Parkinson, S Scheu, and N. Eisenhauer. 2009. Nonlinearity of effects of invasive ecosystem engineers on abiotic soil properties and soil biota. Oikos 118:885-896.

Suárez, E. R., D. M. Pelletier, T. J. Fahey, P. M. Groffman, P. J. Bohlen, and M. C. Fisk. 2004. Effects of exotic earthworms on soil phosphorus cycling in two broadleaf temperate forests. Ecosystems 7:28-44.

Suarez, E. R., T. J. Fahey, P. M. Groffman, J. B. Yavitt, and P. J. Bohlen. 2006. Spatial and temporal dynamics of exotic earthworm communities along invasion fronts in a temperate hardwood forest in South-Central New York (USA). Biological Invasions 8:553-564.

Whitfeld, T. J., A. M. Roth, A. G. Lodge, N. Eisenhauer, L. E. Frelich, and P. B. Reich. 2014. Resident plant diversity and introduced earthworms have contrasting effects on the success of invasive plants. Biological invasions 16:2181-2193.

Wironen, M., and T. R. Moore. 2006. Exotic earthworm invasion increases soil carbon and nitrogen in an old-growth forest in southern Quebec. Canadian Journal of Forest Research 36:845-854.

Yavitt, J. B. 2015. Land use history determines non-native earthworm impacts on atmospheric methane consumption in forest soils, central New York State. Canadian Journal of Soil Science 95:321-330.

Zhang, W., P. F. Hendrix, L. E. Dame, R. A. Burke, J. Wu, D. A. Neher, ... and S. Fu. 2013. Earthworms facilitate carbon sequestration through unequal amplification of carbon stabilization compared with mineralization. Nature Communications 4:2576.
